# Predictive value of neutrophil to lymphocyte and platelet to lymphocyte ratio in COVID-19

**DOI:** 10.1186/s13054-020-03258-x

**Published:** 2020-08-28

**Authors:** Shiping Zhu, Lei Dong, Wanru Cai

**Affiliations:** 1 Respiratory Department, Hangzhou Hospital of Traditional Chinese Medicine, No. 453, Tiyuchang Road, Hangzhou, Zhejiang China; 2 Respiratory Department, The Second Affiliated Hospital of Zhejiang Chinese Medicine University, No. 318, Chaowang Road, Hangzhou, 310000 China

**Keywords:** Neutrophil to leucocyte ratio, Platelet to lymphocyte ratio, COVID-19

To the Editor,

In a recent study, Dr. Ma [1] investigated the neutrophil to lymphocyte ratio (NLR) in predicting moderate-severe acute respiratory distress syndrome (ARDS) in patients with COVID-19 infection. They reported that NLR was significantly higher ARDS group and was a good predictor for moderate-severe ARDS, with area under curve (AUC) 0.749. This study is well designed. However, several limitations should be mentioned. First, uncontrolled inflammatory response plays a vital role in COVID-19 disease, and both NLR and platelet to lymphocyte ratio (PLR) have been recognized as inflammatory factors in various lung diseases [2, 3], such as lung cancer and obstructive lung disease. However, one common limitation is that in most previous studies, NLR or PLR was included in the generalized linear models as a continuous variable, with the assumption that there was a linear association between NLR/PLR and the dependent outcomes. Even in some prediction model [4], researchers only focused on the impact of higher NLR/PLR value while the effect of low NLR/PLR was ignored. However, in most cases, extremely low NLR or PLR represented low neutrophil and platelet, which, according to clinical experience, was also associated with poor outcomes. Thus, the association between NLR/PLR and outcomes should be non-linear, and simply including these parameters into a linear model may lead to a biased conclusion. For instance, we estimated an unadjusted association between NLR/PLR and mortality in critically ill patients with lung infection, using data from the MIMIC database (Fig. 1). Although the cohort is different from the current study, the non-linear association, to a certain degree, supports our hypothesis. If this is the case, the predictive value of NLR/PLR in AUC may also be compromised as both high and low NLR/PLR was associated with increased mortality. The following method may improve this limitation: (1) applying non-linear spline regression using the cut-off value of NLR/PLR in logistic model, (2) generating predictive values of the logistic model, and (3) calculating the AUC of the predictive value in predicting mortality. Finally, we thank Dr. Ma et al. for their valuable research.

**Fig. 1:**
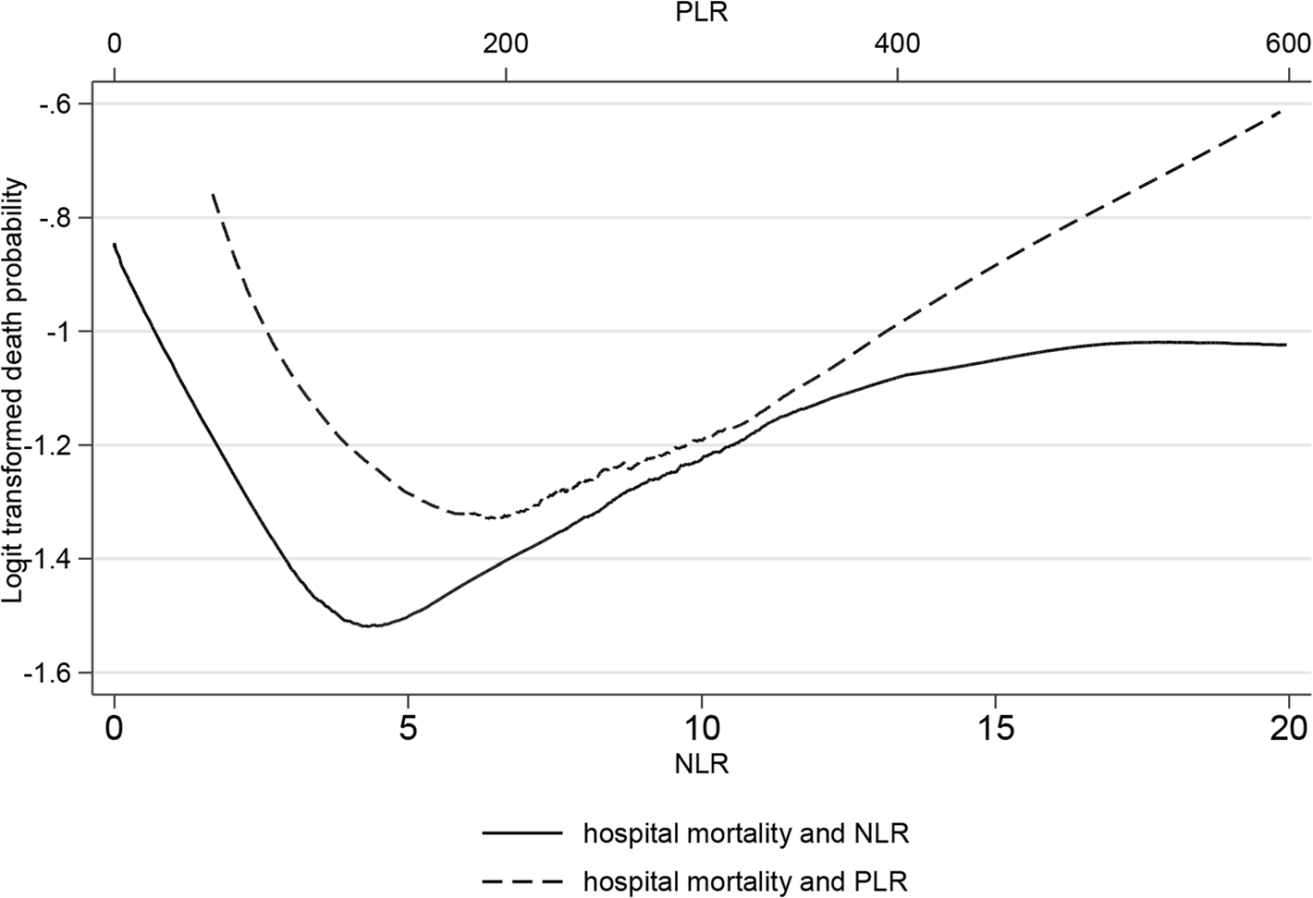
Non-linear association between NLR/PLR and in-hospital death in patients with lung infection. Abbreviations: *NLR* neutrophil to lymphocyte, *PLR* platelet to lymphocyte

## Data Availability

Not applicable.

## Abbreviations

NLR: Neutrophil to lymphocyte ratio
ARDS: Acute respiratory distress syndrome
AUC: Area under curve
PLR: Platelet to lymphocyte ratio
